# Supplementary material for: Transcriptomic Insights into Caffeine Degradation Pathways in Desarmillaria tabescens
Source: Microorganisms. 2025 Nov 28;13(12):2720. doi: 10.3390/microorganisms13122720 (PMC12735256; doi:10.3390/microorganisms13122720)
Supplement: Supplementary file 1 [file microorganisms-13-02720-s001.zip › Supplementary Materials_FigureS1-S6.pdf]

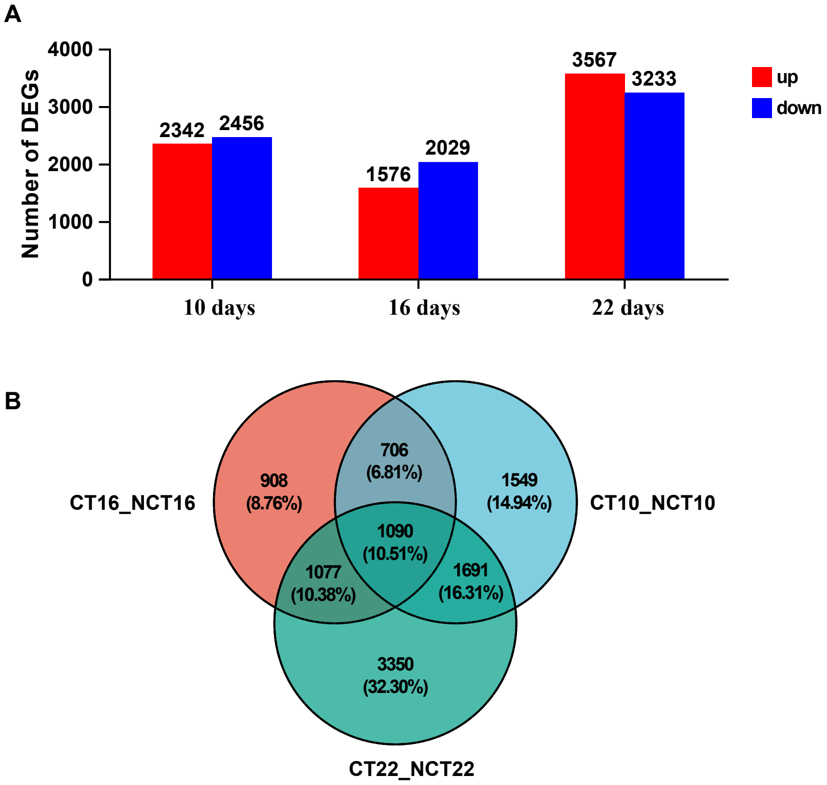

Figure S1 A. Bar chart of DEG counts B. Venn Diagram of DEG Counts

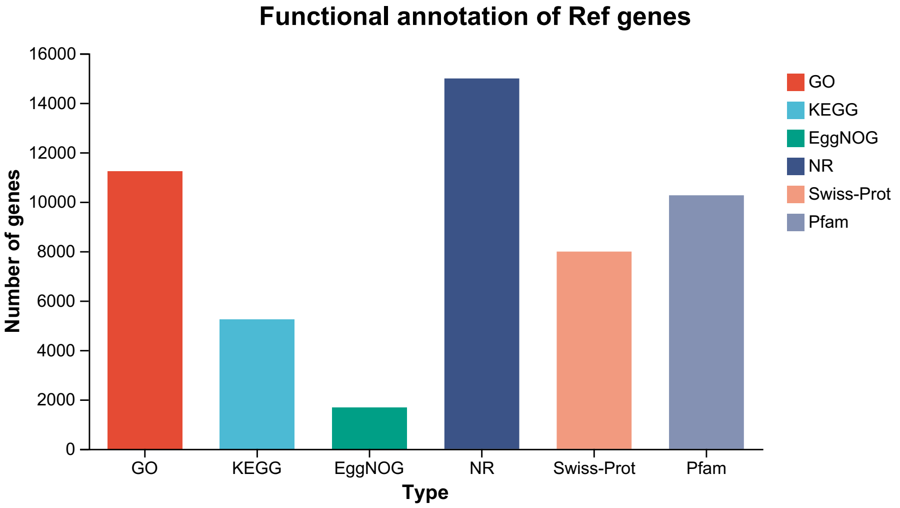

Figure S2 Functional annotation of all assembled genes against six widely used Databases

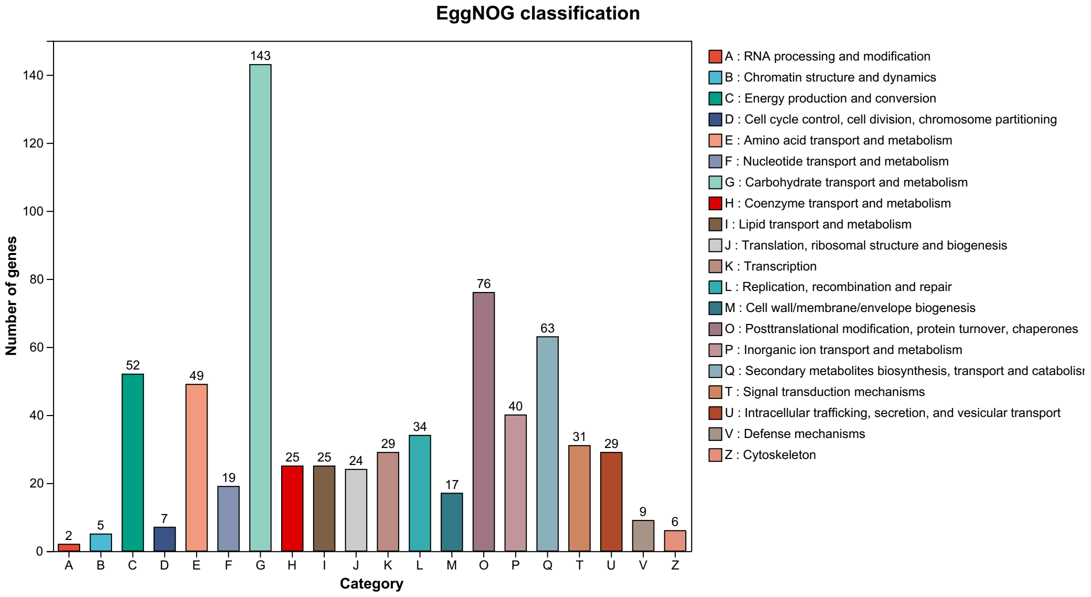

Figure S3 Bar Chart of EggNOG Classification of DEGs. Note: The abscissa represents a functional category of EggNOG (denoted by uppercase letters A-Z; refer to the EggNOG Classification Statistics Table for specific meanings); the ordinate indicates the number of genes/transcripts with this type of function.

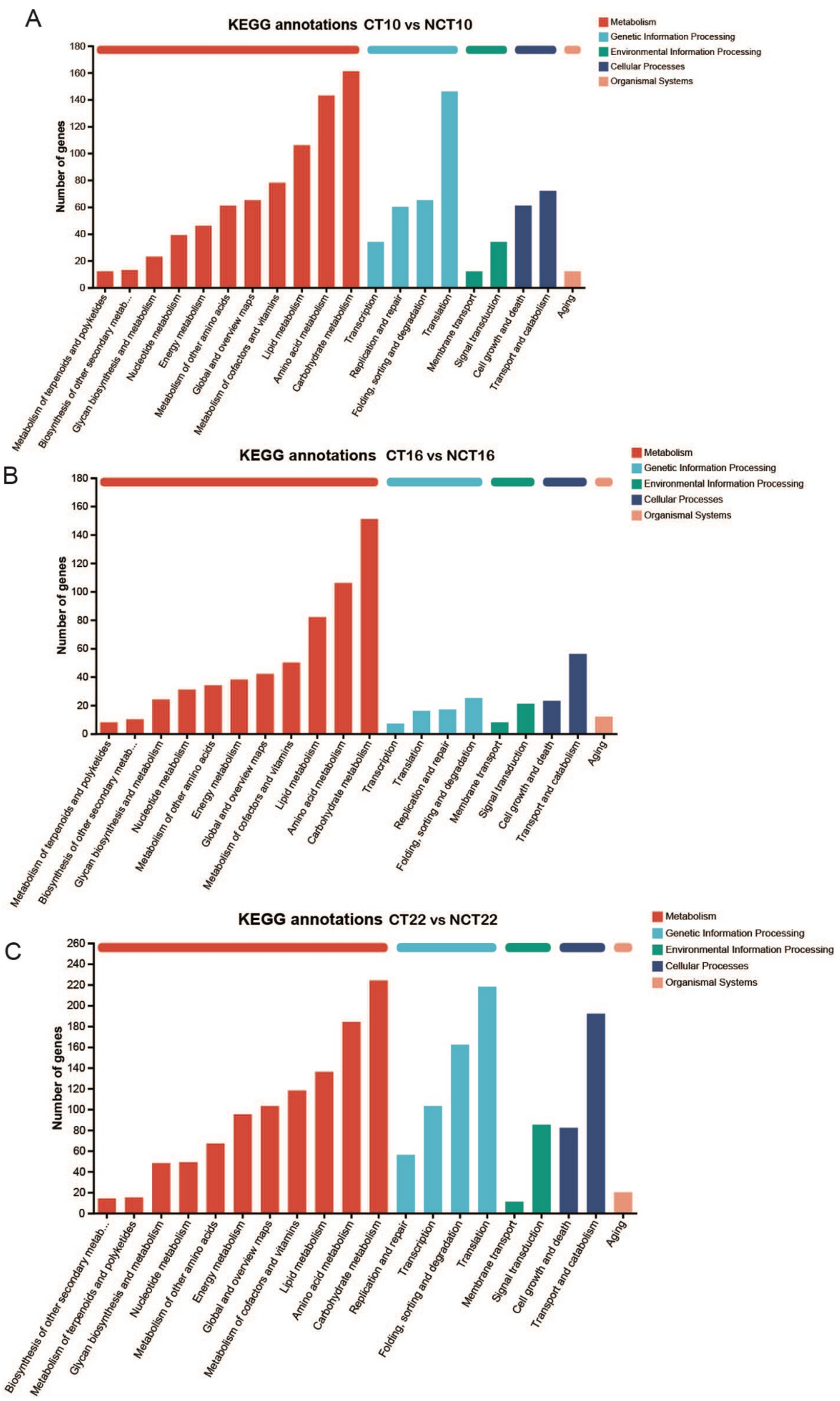

A
KEGG annotations CT10 vs NCT10
Metabolism
Genetic Information Processing
Environmental Information Processing
Cellular Processes
Organismal Systems
Number of genes
180
160
140
120
100
80
60
40
20
0
Metabolism of terpenoids and polyketides
Biosynthesis of other secondary metab...
Glycan biosynthesis and metabolism
Nucleotide metabolism
Energy metabolism
Metabolism of other amino acids
Global and overview maps
Metabolism of cofactors and vitamins
Lipid metabolism
Amino acid metabolism
Carbohydrate metabolism
Transcription
Replication and repair
Folding, sorting and degradation
Translation
Membrane transport
Signal transduction
Cell growth and death
Transport and catabolism
Aging
B
KEGG annotations CT16 vs NCT16
Metabolism
Genetic Information Processing
Environmental Information Processing
Cellular Processes
Organismal Systems
Number of genes
180
160
140
120
100
80
60
40
20
0
Metabolism of terpenoids and polyketides
Biosynthesis of other secondary metab...
Glycan biosynthesis and metabolism
Nucleotide metabolism
Metabolism of other amino acids
Energy metabolism
Global and overview maps
Metabolism of cofactors and vitamins
Lipid metabolism
Amino acid metabolism
Carbohydrate metabolism
Transcription
Translation
Replication and repair
Folding, sorting and degradation
Membrane transport
Signal transduction
Cell growth and death
Transport and catabolism
Aging
C
KEGG annotations CT22 vs NCT22
Metabolism
Genetic Information Processing
Environmental Information Processing
Cellular Processes
Organismal Systems
Number of genes
260
240
220
200
180
160
140
120
100
80
60
40
20
0
Biosynthesis of other secondary metab...
Metabolism of terpenoids and polyketides
Glycan biosynthesis and metabolism
Nucleotide metabolism
Metabolism of other amino acids
Energy metabolism
Global and overview maps
Metabolism of cofactors and vitamins
Lipid metabolism
Amino acid metabolism
Carbohydrate metabolism
Replication and repair
Transcription
Folding, sorting and degradation
Translation
Membrane transport
Signal transduction
Cell growth and death
Transport and catabolism
Aging

Figure S4 KEGG enrichment analyses of DEGs. The x-axis represents the most enriched pathways; the y-axis represents the number of DEGs. A. KEGG annotation of CT10 vs NCT10. A. KEGG annotation of CT16 vs NCT16. A. KEGG annotation of CT22 vs NCT22.

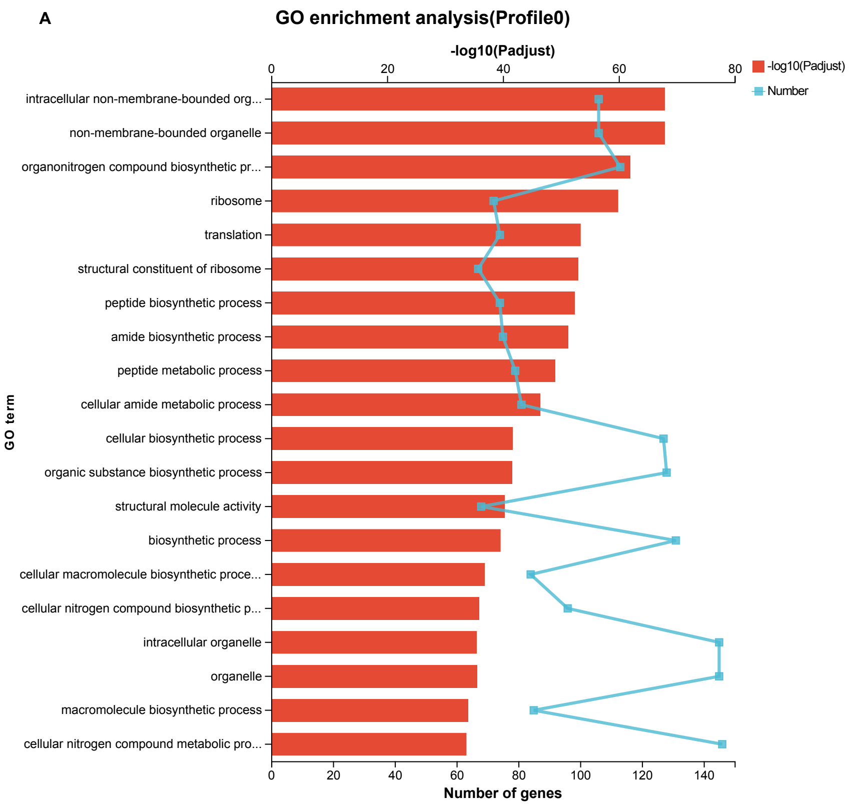
A
GO enrichment analysis(Profile0)
-log10(Padjust)
0
20
40
60
80
-log10(Padjust)
Number
intracellular non-membrane-bounded org...
non-membrane-bounded organelle
organonitrogen compound biosynthetic pr...
ribosome
translation
structural constituent of ribosome
peptide biosynthetic process
amide biosynthetic process
peptide metabolic process
cellular amide metabolic process
cellular biosynthetic process
organic substance biosynthetic process
structural molecule activity
biosynthetic process
cellular macromolecule biosynthetic proce...
cellular nitrogen compound biosynthetic p...
intracellular organelle
organelle
macromolecule biosynthetic process
cellular nitrogen compound metabolic pro...
GO term
0
20
40
60
80
100
120
140
Number of genes

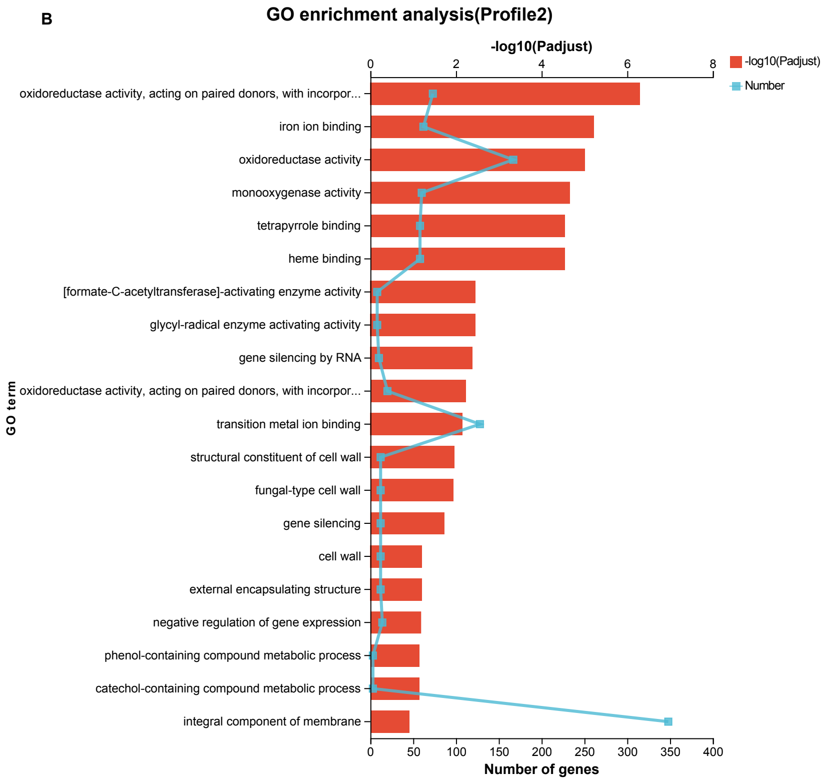
B
GO enrichment analysis(Profile2)
-log10(Padjust)
0
2
4
6
8
-log10(Padjust)
Number
oxidoreductase activity, acting on paired donors, with incorpor...
iron ion binding
oxidoreductase activity
monooxygenase activity
tetrapyrrole binding
heme binding
[formate-C-acetyltransferase]-activating enzyme activity
glycyl-radical enzyme activating activity
gene silencing by RNA
oxidoreductase activity, acting on paired donors, with incorpor...
transition metal ion binding
structural constituent of cell wall
fungal-type cell wall
gene silencing
cell wall
external encapsulating structure
negative regulation of gene expression
phenol-containing compound metabolic process
catechol-containing compound metabolic process
integral component of membrane
GO term
0
50
100
150
200
250
300
350
400
Number of genes

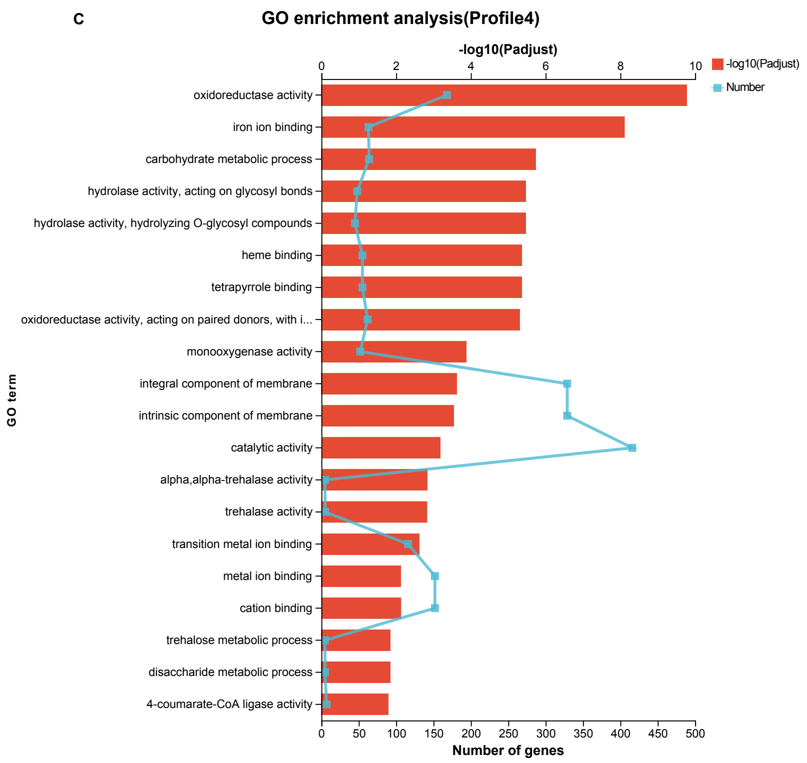

Figure S5 A. GO enrichment analyses of Profile0. B. GO enrichment analyses of Profile2. C. GO enrichment analyses of Profile4.

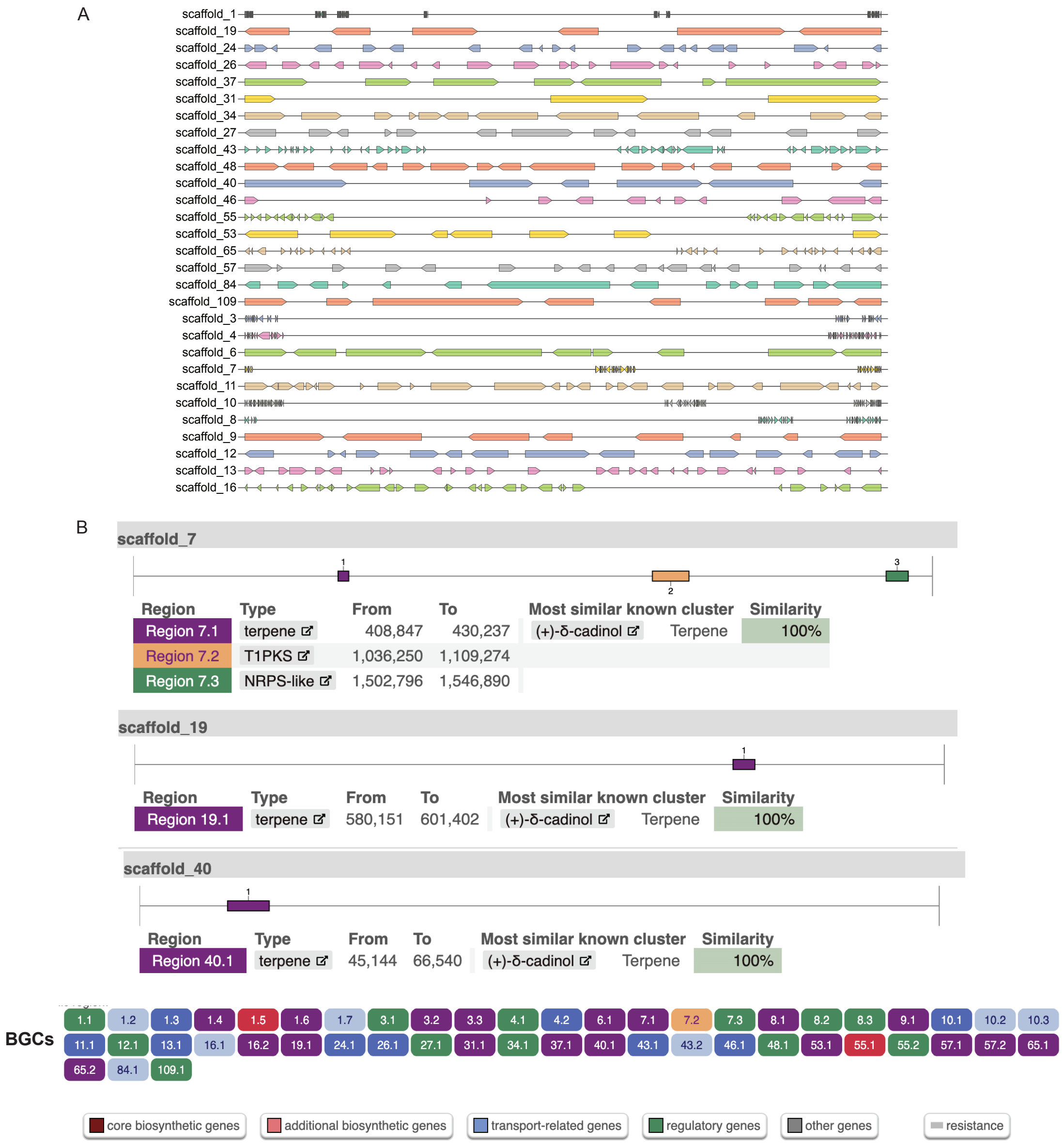

Figure S6 A.AntiSMASH Result of Secondary Metabolite Biosynthetic Gene Clusters B. AntiSMASH results for three regions associated with caffeine degradation.
